# Persistent Neurological Symptoms in Chronic Disease Patients After COVID‐19 Infection in Jamaica: A Retrospective Cohort Study Exploring Clinical Manifestations of Long COVID in a Low and Middle Income Country

**DOI:** 10.1002/hsr2.72761

**Published:** 2026-07-07

**Authors:** Jacqueline P. Duncan, Maria Jackson, Carol Jones Cooper, Joshua J. Anzinger, Kelvin Ehikhametalor, Keri‐Ann Facey, Gavin Cloherty, Marshall K. Tulloch‐Reid

**Affiliations:** ^1^ Department of Community Health and Psychiatry The University of the West Indies Kingston Jamaica; ^2^ Epidemiology Research and Training Unit Kingston Jamaica; ^3^ Department of Microbiology The University of the West Indies Kingston Jamaica; ^4^ Department of Surgery, Radiology Anaesthesia and Intensive Care The University of the West Indies Kingston Jamaica; ^5^ Epidemiology Research Unit, Caribbean Institute for Health Research The University of the West Indies Kingston Jamaica; ^6^ Infectious Diseases Research Abbott Laboratories Abbott Park Illinois USA; ^7^ Abbott Pandemic Defense Coalition Abbott Park Illinois USA

**Keywords:** Caribbean, COVID‐19, Jamaica, long COVID

## Abstract

**Background and Aims:**

There is uncertainty about the long term effects of COVID‐19 infection. We describe the dominant reported symptoms and their sociodemographic risk factors in a sample of Jamaican patients with non‐communicable diseases (NCD) and laboratory determined infection status.

**Methods:**

A retrospective cohort study (February 20, 2023–September 12, 2023) of patients from the University Hospital of the West Indies (UHWI) were identified through participation in the Caribbean COVID‐19 Metabolic Health Study (CCMHS) or ICU admissions between March 2020 and March 2021 with a positive PCR. COVID‐19 infection status was based on anti‐nucleocapsid (anti‐N) antibodies in those without PCR reports. Participants were classified as “Exposed” if they had positive anti‐N antibodies or a positive PCR test and “Unexposed” if they had negative anti‐N antibodies. Bivariate analyses compared current symptoms and quality of life of exposed and unexposed participants. Logistic regression explored factors associated with memory loss.

**Results:**

Eighty‐six persons (62 exposed and 24 unexposed; mean age 54.1 ± 13.4 years; median “time since first COVID‐19 diagnosis” 1.9 years) participated in the study. “Not [feeling] quite right” (59.3%), joint pains (46.5%), fatigue (45.4%), numbness/tingling (43.0%), headache (26.7%), and shortness of breath (23.3%) were the most common symptoms that were similarly reported in persons regardless of infection status. Memory loss was more common among exposed participants (41.9% vs. 12.5%, *p* = 0.01) and among those with COVID‐19 infection, self‐reported depression/anxiety increased the odds of this symptom (OR 6.7 (1.7–25.7).

**Conclusion:**

Cardiopulmonary and musculoskeletal symptoms were common among NCD patients regardless of previous COVID‐19 infection. However, memory loss was more common up to 2 years after initial COVID‐19 infection. Prospective cohorts of long COVID in subpopulations are critical to elucidating its natural history.

## 1 Introduction

Jamaica, a predominantly black, low and middle income country (LMIC) in the Caribbean, identified its first case of coronavirus disease 2019 (COVID‐19) on March 10, 2020. As of January 1, 2024, approximately 156,600 COVID‐19 cases were recorded and excess deaths were estimated at 206 per 100,000 population [1]. During the early stages of the pandemic, Jamaica had limited access to diagnostic testing and depended on shielding, restrictions on movement and gathering in public spaces, to slow the spread of infection. The country experienced several waves of COVID‐19 with the “ancestral” then Alpha variant dominating between March 2020 and June 2021, Delta variant between July 2021 and December 2021 and Omicron variant in subsequent periods (January 2022 to September 2024) [2]. COVID‐19 vaccination was eventually introduced in March 2021, however by December 31, 2022, only 30% of the population had received at least one dose of vaccine [3]. This resulted in a large proportion of the population being vulnerable to COVID‐19 and its long‐term complications such as long COVID.

Post coronavirus disease 2019 (COVID‐19) conditions or long COVID is defined by the WHO as the “continuation or development of new symptoms 3 months after the initial SARS‐CoV‐2 infection, with these symptoms lasting for at least 2 months with no other explanation” [4]. However, other definitions are used in epidemiological studies to characterize long COVID and its risk factors. For example, the United States Department of Health and Human Services defines long COVID as “signs, symptoms, and conditions that continue or develop after initial SARS‐CoV‐2 infection” [5]. Studies have also used a cut‐off of 4 weeks for new or persistent symptoms and objective criteria for COVID‐19 diagnosis such as polymerase chain reaction (PCR) test to define long COVID [6]. Testing for antibodies to nucleocapsid protein (anti‐N antibodies) and/or spike proteins have also been used to identify asymptomatic or mild COVID‐19 [7]. Without a consensus definition and diagnostic tests, designing studies and estimating the prevalence of long COVID is challenging.

WHO estimates that 10% to 20% of persons infected with COVID‐19 will have persistent symptoms. However, studies also show that long COVID prevalence varies with time from COVID‐19 infection, sex, geographic region and severity of presentation [8, 9]. For example, a 2022 meta‐analysis of 41 studies reported higher prevalence in hospitalized compared to non‐hospitalized patients (54% vs 34%), females compared to males (49% vs 27%), and residents in Asia compared to USA (51% vs 31%) [8]. In African countries, female sex, older age, low level of education, severity of acute infection and underlying comorbidity were associated with long COVID [9]. Long COVID prevalence and symptom occurrence also vary with follow up period after acute COVID‐19 [10], COVID‐19 vaccination status [11] and type of SARS‐CoV‐2 variant [12].

Over 200 symptoms, involving several organ systems to varying degrees, have been described in long COVID but the most common symptoms reported are fatigue, cognitive dysfunction, joint pains and shortness of breath [8, 13]. Many persons experience symptoms up to 2 years after COVID‐19 infection [14] and functional impairment/disability resulting in reduced quality of life has been documented [7]. Despite the growing burden of this condition, no specific treatment is available and information on long COVID in LMICs like Jamaica is sparse.

This paper is the first cohort study that we are aware of examining long COVID in the English Speaking Caribbean and describes the dominant symptoms and quality of life of Jamaican patients living with non‐communicable diseases (NCDs) according to COVID‐19 infection status.

## 2 Materials & Methods

A retrospective cohort study was conducted between February 20, 2023 and September 12, 2023.

### 2.1 Study Population

The study population included (i) participants enrolled as part of The Caribbean COVID‐19 Metabolic Health Study (CCMHS) from the outpatient Medical Clinics of the University Hospital of the West Indies (UHWI)—a tertiary referral hospital located in Kingston, Jamaica –as well as (ii) patients with chronic diseases (diabetes mellitus (DM), hypertension (HTN) or hypercholesterolemia) who were admitted to the UHWI Intensive Care Unit (ICU) with COVID‐19 infection between March 1, 2020 and March 31, 2021.

The CCMHS was a cross sectional study, conducted between October 2021, and January 2023, using telephone interviews to evaluate the effect of COVID‐19 control measures on metabolic health in Jamaica, Barbados and Trinidad and recruited persons living with non‐communicable diseases (NCDs) who attended outpatient clinics. Our cohort was restricted to persons from the UHWI in Kingston.

The study was approved by the University of the West Indies Mona Research Ethics Committee (ECP 226, 19/20).

### 2.2 Data Collection

The UHWI patients were first contacted by a medical records officer for consent to be re‐contacted to participate in this study. Participants were then invited to return for a study visit between February 20, 2023 and September 12, 2023. After obtaining written informed consent, a standardized questionnaire was used to collect information on demographics, lifestyle (cigarette, alcohol and marijuana use), past medical history, symptoms at COVID‐19 diagnosis and current symptoms. Quality of life was determined using the Short Form Survey‐36 (SF‐36). Anthropometric measurements (weight, height and waist circumference) were measured using a standard protocol [15].

A non‐fasting blood sample was collected from all participants, and serum was tested for antibodies to nucleocapsid on an ARCHITECT i1000SR instrument. Chemiluminescence immunoassay was conducted using the Abbott ARCHITECT SARS‐CoV‐2 IgG assay for nucleocapsid‐specific IgG. The cutoff ≥ 0.4 signal‐to‐cutoff (S/CO) was used for SARS‐CoV‐2 IgG assays to maximize sensitivity [16].

### 2.3 Variable Definition

Participants were classified as “exposed” and “unexposed” based on their COVID‐19 status.

The following participants were considered “exposed”:

1. Patients from the CCMHS who self‐reported COVID‐19 infection confirmed by positive COVID‐19 test at diagnosis OR
2. Patients from the CCMHS who tested positive for antibodies to the nucleocapsid protein of SARS‐CoV‐2 (anti‐N antibodies) ‐regardless of self‐reported COVID‐19 infection status OR
3. Surviving patients with chronic diseases (specifically diabetes mellitus (DM), hypertension (HTN) or hypercholesterolemia) and a positive COVID‐19 PCR test who were admitted to the ICU at UHWI between March 1, 2020 and March 31, 2021.

### 2.4 Unexposed Participants

Participants with a similar age and sex distribution as the “exposed group” were identified from the list of UHWI CCMHS participants who did not self‐report COVID‐19 infection. These participants had serological tests performed once they came in for evaluation and were re‐classified as “exposed” if anti‐N antibodies were positive and “unexposed” if anti‐N antibodies were negative.

### 2.5 Covid‐19 Experiences

“Time since first COVID‐19 diagnosis” was calculated by subtracting date of first COVID‐19 diagnosis from the date of the study interview.

Persons were asked about previous COVID‐19 infection (Yes or No), number of COVID‐19 infections and whether COVID‐19 testing was done at diagnosis.

Persons were classified as having “severe COVID‐19” if they were hospitalized (including ICU admission) due to COVID‐19. Mild COVID‐19 were exposed persons (as defined above) who were not hospitalized.

Participants were asked if they received COVID‐19 vaccination (Yes or No), number of vaccines and type of vaccines.

### 2.6 Self‐Reported Symptoms

Symptoms were grouped as follows:

1. General symptoms: “yes” response to experiencing fatigue, joint paint, eye discomfort, strong muscle pain, skin rash, chills or fever
2. Cardiopulmonary symptoms: if response was “yes” to experiencing shortness of breath, chest pain, persistent cough, wheezing or hoarseness
3. Neurological symptoms: “yes” response to numbness/tingling, memory problems, headache, dizziness, brain fog, confusion/drowsiness, loss of taste, loss of smell
4. Gastrointestinal symptoms: “yes” response to loss of appetite, nausea or vomiting, abdominal pain or diarrhoea
5. Genito‐urinary symptoms: “yes” response to difficulty holding urine
6. Ear, nose and throat symptoms: “yes” response to earache/tinnitus, or sore throat

Based on our review of the literature significant emphasis was placed on the neurological manifestations that were suggestive of long COVID. Symptoms related to memory loss were determined by “yes” or “no” responses to “Have you experienced any MEMORY RELATED PROBLEMS in the last 2 years?”. Persons who responded “yes” were asked to indicate if there was short‐ term memory loss, long‐term memory loss, not able to remember new things, forgetting routine tasks or other. Short‐term memory loss was defined as the inability to recall recent information such as conversations or events that occurred a short while ago (30 s to a few minutes). Long‐term memory loss was forgetting information/events that occurred over extended periods (hours to years) including “forgetting you've done a task, forgetting recently learned information, or forgetting your third‐grade experience”.

Brain fog was categorized as any of the following: Executive function impairment (difficulty with planning, organizing, figuring out/determining the sequence of actions); Agnosia (failure to recognize or identify objects despite intact sensory functioning), difficulty solving problems or making decisions, difficulty thinking and thoughts moving too quickly.

### 2.7 Sample Size

Sample size for this study was limited by the number of participants in the UHWI arm of the CCMHS study and the UHWI ICU admissions and those identified from the CCMHS of similar age group and sex as the index cases. Preliminary data indicated 58 participants had COVID‐19 infection (38 self‐reported in CCMHS study; 20 ICU admissions) and 61 patients reported no history of COVID‐19 (self‐reported). Antibody testing however, revealed that 62 patients were exposed to COVID‐19 infection and only 24 participants were unexposed.

### 2.8 Statistical Analyses

Analyses were done using STATA Version 14.0. Frequency tables were generated and descriptive analyses used for the different categories of COVID‐19 infection. Means and standard deviations were calculated for normally distributed continuous variables. Bivariate analyses were used to examine differences in symptoms, and quality of life between exposed and unexposed participants. Comparisons of COVID‐19 experiences and symptoms were done based on exposure status and severity of COVID‐19 (severe COVID‐19, mild COVID‐19 vs no COVID‐19). Chi‐square statistic or Fisher's exact test was used for differences in categorical variables. A *p* < 0.05 was considered statistically significant. P values are reported following standard conventions for decimal places e. *p* < 0.001, to the nearest thousandth for p values between 0.001 and 0.01, to the nearest hundredth for *p* values ≥ 0.01, and *p* > 0.99 for *p* values > 0.99). A two‐sided significance level of 0.05 was applied for all tests.

Logistic regression models were used to conduct age‐adjusted analyses and assess associations between variables and memory loss including sociodemographics (sex, employment, education), COVID‐19 severity, and comorbidities (diabetes mellitus, hypertension, obesity, depression/anxiety). Age‐adjusted odds ratios and 95% confidence intervals were generated.

## 3 Results

**Figure 1:**
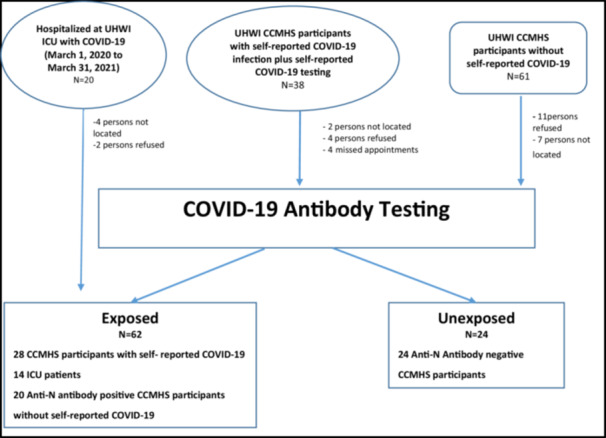
Flow chart of participant selection for retrospective cohort study of long COVID between February 20, 2023 and September 12, 2023, Kingston Jamaica. Note. CCMHS = The Caribbean COVID‐19 Metabolic Health Study; UHWI = University Hospital of the West Indies; ICU = Intensive Care Unit; Anti‐N antibody = antinucleocapsid antibody. Exposed = participants with self‐reported COVID‐19 plus had COVID‐19 test OR anti‐nucleocapsid antibody positive. Unexposed = participants without self‐reported COVID‐19 AND negative anti‐nucleocapsid antibody positive.

We identified 58 possible cases of COVID‐19 infection or exposed persons (38 CCMHS participants that self‐reported COVID‐19 and 20 ICU patients admitted with COVID‐19). Of the 58 exposed persons, 16 were not interested in participating in this study or could not be located. One patient who reported having COVID‐19 infection but who did not have a confirmatory test and also tested negative for antibodies was eventually classified as unexposed (Figure 1).

Sixty‐one unexposed persons (similar age and sex) from the remaining CCMHS participants were approached for the long COVID study. Of the 61 persons selected as potentially unexposed, 18 persons did not return for face‐to‐face visit (refusals, time constraints and inability to locate) while 20 of these persons tested positive for anti‐N antibodies and were therefore classified as exposed (Figure 1). The final sample after consideration of the anti‐N antibody test results consisted of 86 persons, 62 exposed and 24 unexposed.

### 3.1 Characteristics of Study Participants

The mean age of participants was 54.1 ± 13.4 years and median “time since first COVID‐19 diagnosis” was 1.9 years, (IQR 1.4, 2.1 years). Participants' first COVID−19 infection occurred between August 2020 and October 2022 with only 1 participant reporting having their first COVID‐19 infection less than 6 months prior to the study interview date. There were no significant differences in baseline characteristics such as age, sex, body mass index (BMI), current smoking, marijuana use, and comorbidities (except hypercholesterolemia) between exposed and unexposed participants (Table 1).

**Table 1:** Demographic and Clinical characteristics of Exposed and Unexposed Study Participants.

Characteristic	Total participants *N* = 86	Exposed^a^ *N* = 62	Unexposed *N* = 24	*p*‐value
Age ‐ years (mean ± SD)	54.1 ± 13.4	54.0 ± 13.5	54.5 ± 13.6	
Age, *n* (%)				0.54
< 40	16 (18.6)	12 (19.4)	4 (16.7)	
40–59	35 (40.7)	27 (43.6)	8 (33.3)	
≥ 60	35 (40.7)	23 (37.1)	12 (50.0)	
Sex, *n* (%)				0.20
Male	28 (32.6)	23 (37.1)	5 (20.8)	
Female	58 (67.4)	39 (62.9)	19 (79.2)	
Education category (*n* (%))				0.17
Primary school or less	12 (14.0)	8 (12.9)	4 (16.7)	
Secondary/high/vocational school	43 (50.0)	28 (45.2)	15 (62.5)	
College/tertiary	31 (36.1)	26(41.9)	5 (20.8)	
Employment, *n* (%)				0.68
Employed	58 (67.4)	41 (66.1)	17 (70.8)	
Unemployed^b^	28 (32.6)	21 (33.9)	7 (29.2)	
Current smoker, *n* (%)				0.62
Yes	5 (5.8)	3 (4.8)	2 (8.3)	
No	81 (94.2)	59 (95.2)	22 (91.7)	
Current alcohol use, *n* (%)				0.71
Yes	35 (40.7)	26 (41.9)	9 (37.5)	
No	51 (59.3)	36 (58.1)	15 (62.5)	
Current marijuana use, *n* (%)				0.13
Yes	5 (5.8)	2 (3.2)	3 (12.5)	
No	81 (94.2)	60 (96.8)	21 (87.5)	
Body Mass index (kg/m^2^)				0.19
< 25.0	21 (24.4)	12 (19.4)	9 (37.5)	
25.0–29.9	16 (18.6)	13 (21.0)	3 (12.5)	
≥ 30.0	49 (57.0)	37 (59.7)	12 (50.0)	
Comorbidities (*n* (%))				
Obesity or overweight	45 (52.3)	36 (58.1)	9 (37.5)	0.12
Diabetes	32 (37.2)	24 (38.7)	8 (33.3)	0.64
Hypertension	58 (67.4)	44 (71.0)	14 (58.3)	0.26
High cholesterol	37 (43.0)	30 (48.4)	7 (29.2)	0.18
Heart disease	11 (12.8)	10 (16.1)	1 (4.2)	0.17
Depression/anxiety	20 (23.3)	15 (24.2)	5 (20.8)	> 0.99
Other^c^	47 (54.7)	29 (46.8)	18 (75.0)	0.02

^a^ Exposed were anti‐nucleocapsid antibody positive.

^b^ Unemployed includes unemployed persons, retired persons, housewives, and students.

^c^ Other includes rheumatoid and other arthritis (10), SLE (7), thyroid disorder (6), sinusitis (5), anemia (4), other endocrine disorders (2), polymyositis (1), nerve pain (2), psoriasis (2), abnormal uterine bleed (2), glaucoma (2), bipolar disorder (1), central venous thrombosis (1), migraine (1), chronic urticarial (1).

### 3.2 COVID‐19 Experiences by Severity of COVID‐19 Disease

Table 2 summarizes COVID‐19 experiences by severity of illness. Longer duration of illness was more common among persons with history of severe COVID‐19 compared to mild COVID‐19 (*p* < 0.001). More than one‐third of participants with self‐reported COVID‐19 (38.1%) had “extremely bad” symptoms during their first COVID‐19 diagnosis and this was also more common for hospitalized participants or severe COVID‐19 (75.0% vs 15.4%, *p* < 0.001). Self‐reported repeat COVID‐19 infection was rare (7.1%) and 50% of severely infected persons did not report being vaccinated. Except for symptom severity and duration, COVID‐19 experiences were similar for severe and mild COVID‐19 cases.

**Table 2:** COVID‐19 Experiences by Severity of Disease.

	Total exposed^a^ *N* = 62	Severe COVID‐19^b^ *N* = 46	Mild COVID‐19^c^ *N* = 16	*p*‐value
Median time since first COVID‐19 diagnosis^d^ (IQR)‐years	1.9 (1.4, 2.1)	2.0 (1.6, 2.3)	1.7 (1.2, 2.1)	
Duration of illness, *n*/*N* e (%)				< 0.001
< 2 weeks	21/42 (50.0)	2/16 (12.5)	19/26 (73.1)	
≥ 2 weeks	21/42 (50.0)	14/16 (87.5)	7/26 (26.9)	
Symptoms at worst, *n*/*N* e (%)				< 0.001
A little/not bad	20/42 (47.6)	3/16 (18.8)	17/26 (65.4)	
Quite/very bad	6/42 (14.3)	1/16 (6.3)	5/26 (19.2)	
Extremely bad	16/42 (38.1)	1/162 (75.0)	4/26 (15.4)	
History of repeat (> 1) COVID‐19 infections, *n*/*N* e (%)	3/42 (7.1)	2/16 (12.5)	1/26 (3.9)	0.55
Anti‐N antibodies, *n* (%)	45 (72.6)	13 (81.3)	32 (70.0)	0.52
Vaccination status at time of interview, (%)				0.13
0	32.3	50.0	26.1	
1	9.7	12.5	8.7	
≥ 2	58.1	37.5	65.2	

^a^ Exposed = participants with history of COVID‐19 plus had COVID‐19 test **OR** Anti‐nucleocapsid antibody positive.

^b^ Severe COVID‐19 = “exposed” PLUS hospitalization OR admitted to ICU.

^c^ Mild COVID‐19 = “exposed” but not hospitalized or admitted to ICU.

^d^ Time since first COVID‐19 diagnosis = date of the study interview minus date of first COVID‐19 diagnosis.

^e^ N = participants who self‐reported COVID‐19 infection (*N* = 42).

### 3.3 Self‐Reported Health Status and Symptoms Experienced by COVID‐19 Exposure Status

Neurological (68.6%), cardiopulmonary (46.5%), and gastrointestinal (24.4%) symptoms were commonly experienced by both exposed and unexposed participants (Table 3). Many participants (59.3%) reported that they were feeling “not quite right” and general symptoms such as fatigue, muscle or joint pains were common (77.9% participants). The most frequently reported symptoms were joint pains (46.5%), mild fatigue (45.4%) numbness/tingling (43%), memory problems (33.3%), headache (26.7%), difficulty holding urine (23.3%), unusual shortness of breath (23.3%) and dizziness (20.9%) (Table 3). However, except for memory problems (41.9% vs. 12.5%, *p* = 0.011), there were no significant differences in symptoms between exposed and unexposed participants.

**Table 3:** Current Health Status and Symptoms Experienced by COVID‐19 Exposure Status.

Current symptoms	Total participants *N* = 86	Exposed^a^ *N* = 62	Unexposed *N* = 24	*p*‐value
Self‐reported Health, *n* (%)				0.11
Healthy	35 (40.7)	22 (35.5)	13 (54.2)	
Not quite right	51 (59.3)	40 (64.5)	11 (45.8)	
General symptoms, *n* (%)	67(77.9)	48 (77.4)	19 (79.2)	0.86
Fatigue	43 (50.0)	32 (51.6)	11 (45.9)	0.92
Joint pain	40 (46.5)	29 (46.8)	11 (45.8)	0.94
Eye discomfort	29 (33.7)	20 (32.3)	9 (37.5)	0.80
Strong muscle pain	19 (22.1)	14 (22.6)	5 (20.8)	> 0.99
Skin rash	7 (8.1)	4 (6.5)	3 (12.5)	0.39
Chills or Shivers	3 (3.5)	3 (4.2)	0 (0.0)	0.56
Cardiopulmonary symptoms, *n* (%)	40 (46.5)	32 (51.6)	8 (33.3)	0.13
Shortness of breath	24 (27.9)	20 (32.2)	4 (16.7)	0.26
Chest pain	17 (19.8)	15 (24.2)	2 (8.3)	0.13
Persistent cough	14 (16.3)	11 (17.4)	3 (12.5)	0.75
Wheezing	10 (11.6)	9 (14.5)	1 (4.2)	0.27
Hoarseness	8 (9.3)	7 (11.3)	1 (4.2)	0.43
Neurological symptoms, *n* (%)	59 (68.6)	43 (69.4)	16 (66.7)	0.81
Numbness/tingling	37 (43.0)	27 (43.6)	10 (41.7)	> 0.99
Memory problems	29 (33.7)	26 (41.9)	3 (12.5)	0.01
Headache	23 (26.7)	16 (25.8)	7 (29.2)	0.75
Dizziness	18 (20.9)	13 (21.0)	5 (20.8)	> 0.99
Brain fog	12 (14.0)	11 (17.7)	1 (4.2)	0.17
Confusion/drowsiness	9 (10.5)	8 (12.9)	1 (4.2)	0.43
Loss of taste	4 (4.7)	4 (6.5)	0 (0.0)	0.57
Loss of smell	3 (3.5)	3 (4.2)	0 (0.0)	0.56
Gastrointestinal symptoms, *n* (%)	21 (24.4)	16 (25.8)	5 (20.8)	0.78
Loss of appetite	11 (12.8)	7 (11.3)	4 (16.7)	0.49
Nausea or vomiting	7 (8.1)	5 (8.1)	2 (8.3)	> 0.99
Abdominal pain	7 (8.1)	6 (9.7)	1 (4.2)	0.67
Diarrhoea	5 (5.8)	5 (8.1)	0 (0.0)	0.32
Genitourinary symptoms. *n* (%)				
Difficulty holding urine	20 (23.3)	14 (22.6)	6 (25.0)	0.81
Ear, nose and throat. *n* (%)	15 (17.4)	11 (17.7)	4 (16.7)	> 0.99
Earache/tinnitus	12 (14.0)	8 (12.9)	4 (16.7)	> 0.99
Sore throat	3 (3.5)	3 (4.2)	0 (0.0)	0.56
Other^b^	27 (31.4)	20 (32.3)	7 (29.2)	0.78

^a^ Exposed = participants with history of COVID‐19 plus had COVID‐19 test **OR** Anti‐nucleocapsid antibody positive.

^b^ Other includes joint or muscle spasm or weakness (8), swollen ankles/feet (3), sinusitis/congestion (3), constipation/infrequent stool (2), nerve pain/sciatica (2), erectile dysfunction (2), abnormal heart beat (2), nosebleed (1), generalized itching (1), stomach pain (1), tremor (1) breathing problem (1).

### 3.4 Memory Loss and Brain Fog

About one‐third of participants reported memory problems with the most common memory problems being short‐term (27.9%) memory loss. Both short and long‐term memory loss were at least four times more common for exposed participants compared to the unexposed (Table 4). Fourteen percent of persons (14.0%) reported brain fog and was similar among exposed and unexposed participants. Among those reporting brain fog, poor attention (66.7%), difficulty solving problems (50.0%), difficulty thinking (33.3%), slowed thoughts (33.3%), and impairment of executive function (33.3%) were the most common presentations (Table 4).

**Table 4:** Memory Loss and Brain Fog by COVID‐19 Exposure Status.

Symptom	Total participants *N* = 86	Exposed^a^ *N* = 62	Unexposed *N* = 24	*p*‐value
Memory problems	29 (33.7)	26 (41.9)	3 (12.5)	0.01
Short term memory loss^b^ – *n* (%)	24 (27.9)	22 (35.5)	2 (8.3)	0.02
Long term memory loss^c^ – *n* (%)	11 (12.8)	11 (7.4)	0 (0.0)	0.03
Not remembering new things – *n* (%)	5 (5.8)	5 (8.1)	0 (0.0)	0.32
Forget routine tasks – *n* (%)	0 (0.0)	0 (0.0)	0 (0.0)	n/a
Others – *n* (%)	3 (3.5)	2 (3.2)	1 (4.2)	> 0.99
Brain Fog	12 (14.0)	11 (17.7)	1 (4.2)	0.17
Executive function impairment^d^– *n* (%)	4 (4.7)	3 (4.8)	1 (4.2)	> 0.99
Agnosia – *n* (%)	0 (0.0)	0 (0.0)	0 (0.0)	n/a
Difficulty solving problems or making decision– *n* (%)	6 (7.0)			
Difficulty thinking– *n* (%)	4 (4.7)	3 (4.8)	1 (4.2)	> 0.99
Thoughts moving too quickly– *n* (%)	2 (2.4)	2 (3.2)	0 (0.0)	> 0.99
Slowed thoughts– *n* (%)	4 (4.7)	4 (6.5)	0 (0.0)	0.57
Poor attention or concentration– (%)	8 (9.3)	8 (12.9)	0 (0.0)	0.10

^a^ Exposed = participants with history of COVID‐19 plus had COVID‐19 test **OR** Anti‐nucleocapsid antibody positive.

^b^ Short‐term memory loss (memory that lasts ~30 s, i.e. remembering a phone number before writing it down, or forgetting you're in the middle of a task).

^c^ Long‐term memory loss (long‐term memory can be anything from remembering yesterday, forgetting you've done a task, forgetting recently learned information, or forgetting your third‐grade experience).

^d^ Executive functioning refers to difficulty with planning, organizing, figuring out the sequence of actions.

Table 5 displays memory loss by socio‐demographic, comorbidities, and severity of COVID 19. Among exposed persons, memory loss was significantly more common among persons reporting depression or anxiety and did not vary by sociodemographic factors, weight status or severity of COVID‐19 disease.

**Table 5:** Memory Loss by Socio‐demographics, Severity of COVID 19, and Comorbidities.

Characteristic	Total participants *N* = 62	Memory loss *N* = 26	No memory loss *N* = 36	*p*‐value
Age, *n* (%)				0.85
< 60 years	39 (62.9)	16 (61.5)	23 (63.9)	
≥ 60 years	23 (37.1)	10 (38.5)	13 (36.1)	
Sex, *n* (%)				0.16
Male	23 (37.1)	7 (26.9)	16 (44.4)	
Female	39 (62.9)	19 (73.1)	20 (55.6)	
Education category (*n* (%))				0.11
Primary/secondary/high/vocational school	36 (58.1)	12 (46.2)	24 (66.7)	
College/tertiary	26 (41.9)	14 (53.9)	12 (33.3)	
Employment, *n* (%)				0.33
Employed	21 (33.9)	19 (73.1)	22 (61.1)	
Unemployed^a^	41 (66.1)	7 (26.9)	14 (38.9)	
Body mass index (kg/m^2^)				0.80
< 30.0	25 (40.3)	10 (38.5)	15 (41.7)	
≥ 30.0	37 (59.7)	16 (61.5)	21 (58.3)	
Comorbidities (*n* (%))				
Depression/anxiety	15 (24.2)	11 (42.3)	4 (11.1)	0.007
Diabetes	24 (38.7)	8 (30.8)	16 (44.4)	0.28
Hypertension	44 (71.0)	18 (69.2)	26 (72.2)	0.80
Severity^b^ (*n* (%))				0.86
Severe	16 (25.8)	7 (26.9)	9 (25.0)	
Mild	46 (74.2)	19 (73.1)	27 (75.0)	

^a^ Unemployed, housewife, disabled.

^b^ Severe COVID‐19 refers to hospitalization including ICU admission.

### 3.5 Quality of Life

Approximately 30% of participants had slight or moderate difficulty walking and 53% experienced slight or moderate pain/discomfort but few participants had problems with self‐care (10.5%). However, there were no significant differences in, quality of life based on exposure status (See supplemental table S1).

Table 6 shows age‐adjusted associations with memory loss among participants with confirmed COVID‐19 infection. Depression/anxiety increased the odds of memory loss (OR 6.7 (1.7–25.7); *p* = 0.006). There was no significant association between memory loss and the socio‐demographic factors examined or severity of COVID‐19 based on the need for hospitalization.

**Table 6:** Odds Ratios (OR) and 95% CI for Association with Memory Loss.

	Age adjusted^a^ OR (95%CI)	*p* value
Sex, *n* (%)		
Female	Reference	0.73
Male	0.5 (0.2–1.4)	
Education category (*n* (%))		
Primary/secondary/high/vocational school	Reference	0.11
College/tertiary	2.3 (0.8–6.6)	
Employment, *n* (%)		
Unemployed^b^	Reference	0.24
Employed	2.1 (0.6–7.2)	
Body mass index (kg/m^2^)		
< 30.0	Reference	0.74
≥ 30.0	1.2 (0.4–3.6)	
cSeverity (*n* (%))		
Severe	1.1 (0.4–3.5)	0.85
Mild	Reference	
Comorbidities (*n* (%))		
No Depression/anxiety	Reference	0.006
Depression/anxiety	6.7 (1.7–25.7)	
No Hypertension	Reference	0.73
Hypertension	0.8 (0.2–2.7)	
No Diabetes Mellitus	Reference	0.26
Diabetes Mellitus	0.5 (0.2–1.6)	

^a^ Memory loss was the outcome variable.

^b^ Unemployed, housewife, disabled.

^c^ Severe COVID‐19 refers to hospitalization including ICU admission.

## 4 Discussion

We are unaware of any publication on long COVID (also called Post‐Acute Sequelae of COVID‐19 or post COVID‐19 condition) in a Caribbean country. Additionally this is one of few studies on long COVID in persons with NCDs. We found that persons with NCDs had a high prevalence of cardiopulmonary, neurological and general symptoms regardless of exposure to COVID‐19. Memory loss (short‐term and long‐term) was the only symptom that was significantly more common after COVID‐19 infection when objective measurement was used for classification of exposure (i.e. positive anti‐N antibody). However, when only self‐reported COVID‐19 was used to determine exposure, symptoms such as chest pain, wheezing, confusion/drowsiness and brain fog were more commonly reported after COVID‐19 infection. This may be due to recall or response bias as persons may be more likely to report symptoms when they are aware that they had COVID‐19, but it may also be due to differences in persistent symptoms after symptomatic COVID‐19.

Generally, studies on long COVID report high prevalence of persistent cardiopulmonary, musculoskeletal, gastrointestinal and neurological symptoms [13]. A 2023 meta‐analysis of 36 studies including 11,598 long COVID patients showed that the most common symptoms of long COVID were fatigue (29.2%; 95% CI 21.6–39.5), joint pain (28.3%; 95% CI 14.8–54.1), anxiety/depression (23.5%; 95% CI 19.8–27.8), dyspnea (21.5%; 95% CI 14.4–32.1), hair loss (20.3%; 95% CI10.6–39.0), memory deficits (18.4% 95%CI 11.7–28.9) and myalgia (13.3%; 95% CI 7.45–23.7) [13]. The median follow‐up time for the studies in this meta‐analysis was 8 months. Sleep disorders, depression and anxiety were also frequently reported in long COVID [17]. More severe COVID‐19 disease, female sex, older age, obesity and other pre‐existing conditions are associated with higher risk of long COVID [8, 17, 18]. We found similarly high cardiopulmonary and musculoskeletal symptom prevalence for both exposed and unexposed participants – which may possibly be explained by our study population being older (mean age 54.1 years) and living with NCDs.

Prevalence of long COVID is shown to decline with time [14, 19]. Most post‐COVID studies were conducted within 1 year after COVID‐19 infection and this influences the prevalence and type of symptoms reported after COVID‐19 infection. A longitudinal prospective study of 3038 participants with mean follow‐up of 26 months after COVID‐19 infection found that 46% participants recovered from fatigue and 57% recovered from cognitive deficits [14]. Cai et al found that 68.5% of 856 Chinese patients with long COVID had resolution of their symptoms at 1 year [20]. However, many persons still experience symptoms up to 2 years after COVID‐19 infection, particularly after severe COVID‐19 [14]. The median follow‐up period in our study was 1.9 years after first COVID‐19 diagnosis hence we anticipated that persistent symptoms after COVID‐19 infection would be less than recorded in other studies. Few studies describe long COVID 2 years or more after COVID‐19 infection and more information is needed on the long‐term consequences of COVID‐19 infection.

Although there were no significant differences in most symptoms experienced by exposed and unexposed participants in our study, short‐term and long‐term memory loss were significantly more common in exposed participants more than 1 year after COVID‐19 infection. Persistent neurological symptoms such as memory loss and concentration difficulties 1 to 2 years after COVID‐19 infection are also frequently reported in studies [18, 21, 22]. Depression, anxiety, severe COVID‐19, length of admission, and socio‐demographics (e.g. female sex and fewer years of education) were associated with cognitive deficits after COVID‐19 infection while COVID‐19 vaccination was shown to attenuate cognitive deficits [22, 23, 24]. We did not assess the impact of vaccination on memory loss as vaccination occurred after first COVID‐19 infection for most participants. Additionally given the large number of subclinical infections identified from our antibody studies it was not possible to determine if vaccination took place before infection. In our study only depression/anxiety, but not sociodemographic characteristics, severity of COVID‐19, diabetes and hypertension, were associated with memory loss. We may however have been underpowered to demonstrate some of these associations in our sample. Additionally, memory loss was based on self‐report and not objective cognitive testing.

With the exception of memory loss, we did not find differences in self‐reported symptoms between exposed and unexposed participants. This may be due to the circulating variants of concern (VOC) and vaccination against COVID‐19. Omicron and epsilon variants are associated with lower risk of long COVID compared to Delta and Alpha variants [25, 26]. However, the prevalence of long COVID may be higher during the Omicron waves due to its high transmissibility and the amplitude of the surge [26]. The type of persistent symptoms may also vary with variant of infection [12]. Min Du et al [12] showed that pooled estimates of sleep difficulty was lowest after infection with Delta variant compared to Omicron while myalgia was highest among persons infected with Omicron variant. Our study participants were interviewed between February and September 2023. Omicron was the dominant circulating VOC in the Caribbean from early 2022 but surges associated with Alpha and Delta variants occurred prior to 2022 [27]. Therefore, our study participants may have experienced COVID‐19 infection with multiple and/or different variants, potentially influencing our study findings. In earlier studies, participants were less likely to have repeat infections and infected persons were less likely to be misclassified as unexposed.

A meta‐analysis examining the effect of vaccination on long COVID symptoms showed that full vaccination was associated with a 40% reduction in long COVID, 40% reduction in persistent fatigue and 50% reduction in pulmonary disorders compared to no vaccination [11]. Jamaica was the first country in the Caribbean to receive vaccines through the COVAX facility in May 2021 but has the second lowest COVID‐19 vaccination coverage in the Caribbean, second to Haiti [28]. To date, approximately 30% of the population is fully vaccinated [3]. Vaccination against COVID‐19 in our study population was significantly higher than levels reported for the general population and similar to vaccination coverage reported in other studies for persons with NCDs (70% to 80%) [29, 30]. Of note, most participants in our study were vaccinated after their first self‐reported COVID‐19 infection and it was therefore not feasible to explore the association between vaccination and memory loss. Vaccination coverage was similar for exposed and unexposed participants.

The role of ethnicity and social determinants of health on long COVID is unclear. Jamaica has a predominantly black population and a 2024 meta‐analysis suggests that Asian and Black races may be associated with a decreased risk of long COVID [31, 32]. Lower educational level and unemployment have also been associated with non‐recovery from COVID‐19 [14, 31]. Further studies on long COVID in the Caribbean and among ethnic minorities are needed to better understand the role of socio‐cultural determinants and to describe long COVID in these subpopulations.

Our study also illustrates challenges with using existing definitions and designing studies to understand long COVID. Establishing previous COVID‐19 infection to identify exposed versus unexposed participants can be fraught with errors. Persons with asymptomatic and mild COVID‐19 may be undiagnosed particularly in low resource settings with limited testing [33]. This may result in misclassification of exposure or COVID‐19 infection. This could have resulted in an underestimation of prevalence of long COVID symptoms. We used a combination of self‐report, COVID‐19 diagnostic test (PCR or rapid antigen test) and test for anti‐N antibodies to reduce misclassification. High levels of anti‐N antibodies (88%) have been found in persons with long COVID 90 days after COVID‐19 infection [34]. However, anti‐N antibodies are more likely to be positive for persons with severe presentation compared to mild and asymptomatic exposed which we were especially interested in identifying [35]. Of note, 68% persons who self‐reported COVID‐19 (confirmed by COVID‐19 test) in our study were negative for anti‐N antibodies (mean follow up 1.7 years after COVID‐19 diagnosis). It is possible that exposed persons did not mount an antibody response or antibodies became undetectable. These findings are consistent with studies that show waning antibodies to nucleocapsid and spike proteins after COVID‐19 infection as well as higher antibody titers after severe COVID‐19 [36, 37].

Misclassification of severe COVID‐19 is less likely based on our definition (hospitalized or ICU admission during first COVID‐19 infection) since all exposed participants that were hospitalized had COVID‐19 testing. However, participants may have been hospitalized during later COVID‐19 infections and these were not classified as severe COVID‐19. Additionally, we did not account for the type of COVID‐19 variant in our analyses. Larger deficits in memory loss have been associated with earlier SARS‐CoV2 variants [22]. Time or date of infection has been used as a proxy for determining variants involved [22, 38] but our use of anti‐N antibodies captured undiagnosed persons whose date of infection could not be ascertained.

Attributing current symptoms to previous COVID‐19 infection can also be problematic. Most studies rely on self‐reported symptoms 4 weeks or more after COVID‐19 infection without objective clinical assessment. We found that cardiopulmonary, musculoskeletal and neurological symptoms, except for memory loss, were similar for exposed and unexposed participants. Studies on long COVID often do not determine pre‐COVID health and lack valid comparison groups. We recruited persons in a health care setting who may have had symptoms or complications due to their NCDs prior to their COVID‐19 diagnosis. Studies show that fatigue and joint pains are frequently reported in older persons, regardless of chronic NCD occurrence [39, 40]. Kawano et al [41] showed that 85% of persons living with diabetes surveyed in Japan had neurological symptoms of which half were not due to diabetic neuropathy. It is possible that COVID‐19 aggravated or worsened these baseline symptoms but this was not captured in the study. Future studies on long COVID should consider pre‐COVID health and functionality when attempting to attribute symptoms to long COVID.

### 4.1 Strengths and Limitations

Despite the small sample our study adds to the body of literature on long COVID as the study was conducted in persons with NCDs in a LMIC with a predominantly black population. Long COVID or post‐COVID condition has not been documented in a similar population and few studies have reported symptoms 2 years after COVID‐19 infection. We purposely included admissions to ICU in order to capture post‐COVID conditions associated with the spectrum of illness severity caused by COVID‐19. The inclusion of exposed and unexposed groups with antibody testing for COVID‐19 strengthens the findings of our study. However, the small sample size and convenience sampling in a hospital setting (medical clinic and ICU admissions) limits generalizability. Selection and survivor bias may have also occurred due to the inclusion of patients that survived ICU admission. Recall bias may have occurred for participants that self‐reported COVID‐19. Despite the use of antibody tests, misclassification of COVID‐19 cases could have also contributed to some of our findings.

## 5 Conclusion

Cardiopulmonary and gastrointestinal symptoms were common among Jamaican patients with NCDs regardless of previous COVID‐19 infection. Memory loss was more common more than 1 year after COVID‐19 infection among persons with NCDs and was associated with anxiety/depression. Larger prospective cohort studies with periodic measurement of the symptoms and evaluation of infection status would be required to improve our understanding of COVID‐19 infection and its natural history. We, however, hope that there will never be another occasion for this type of study in the region.

## Author Contributions

**Jacqueline P. Duncan:** conceptualization, investigation, funding acquisition, writing – original draft, methodology, validation, visualization, writing – review and editing, software, formal analysis, data curation, supervision, resources, project administration. **Maria Jackson:** conceptualization, investigation, funding acquisition, writing – original draft, methodology, validation, visualization, writing – review and editing, formal analysis, project administration, data curation, supervision, resources.

## Ethics Statement

The study was approved by the University of the West Indies Mona Research Ethics Committee (ECP 226, 19/20) on August 26, 2020. Study participants provided written informed consent.

## Conflicts of Interest

Dr. Azinger has a collaborative research agreement with Abbott Laboratories and receives support and an honorarium from Abbott Laboratories. Dr. Cloherty is an employee and shareholder of Abbott Laboratories. Dr. Duncan is a consultant to Omega Research Group (Florida, USA).

## 1 Transparency Statement

The lead author Jacqueline Duncan affirms that this manuscript is an honest, accurate, and transparent account of the study being reported; that no important aspects of the study have been omitted; and that any discrepancies from the study as planned (and, if relevant, registered) have been explained. All authors have read and approved the final version of the manuscript. Jacqueline Duncan had full access to all of the data in this study and takes complete responsibility for the integrity of the data and the accuracy of the data analysis.

## Supporting information


**Table S1:** Quality of Life by COVID‐19 infection.

## Data Availability

The original contributions presented in the study are included in the article. Further inquiries can be directed to the corresponding author.
