# Supplementary material for: Persistent Neurological Symptoms in Chronic Disease Patients After COVID‐19 Infection in Jamaica: A Retrospective Cohort Study Exploring Clinical Manifestations of Long COVID in a Low and Middle Income Country
Source: Health Sci Rep. 2026 Jul 7;9(7):e72761. doi: 10.1002/hsr2.72761 (PMC13341942; doi:10.1002/hsr2.72761)
Supplement: Supplementary file 1 — Table S1: Quality of Life by COVID‐19 infection. [file HSR2-9-e72761-s001.docx]

**Table S1: Quality of Life by COVID-19 infection**

| **Condition** | **Total Participants N=86** | **Exposed^a^**  **N=62** | **Unexposed**  **N=24** | ***P*-value** |
| --- | --- | --- | --- | --- |
| **Quality of life** |  |  |  |  |
| **Mobility, n(%)** |  |  |  | 0.63 |
| no problems walking | 54(62.8) | 38(61.3) | 16(66.7) |  |
| slight problems walking | 20(23.3) | 16(25.8) | 4(16.7) |  |
| moderate problems walking | 10(11.6) | 6(9.7) | 4(16.7) |  |
| severe problems walking | 2(2.3) | 2(3.2) | 0(0.0) |  |
| unable to walk | 0(0.0) | 0(0.0) | 0(0.0) |  |
| **Self Care, n(%)** |  |  |  | 0.83 |
| no problems | 77(89.5) | 54(87.1) | 23(95.8) |  |
| slight problems | 7(8.1) | 6(9.7) | 1(4.2) |  |
| moderate problems | 1(1.2) | 1(1.6) | 0(0.0) |  |
| severe problems | 0(0.0) | 0(0.0) | 0(0.0) |  |
| Unable to wash or dress | 1(1.2) | 1(1.6) | 0(0.0) |  |
| **Usual activities, n(%)** |  |  |  | 0.79 |
| No problems doing usual activities | 59(68.6) | 41(66.1) | 18(75.0) |  |
| slight problems | 20(23.3) | 15(24.2) | 5(20.8) |  |
| moderate problems | 6(7.0) | 5(8.1) | 1(4.2) |  |
| severe problems | 1(1.2) | 1(1.6) | 0(0.0) |  |
| unable to do usual activities | 0(0.0) | 0(0.0) | 0(0.0) |  |
| **Pain or discomfort, n(%)** |  |  |  | 0.63 |
| No pain or discomfort | 24(27.9) | 18(29.0) | 6(25.0) |  |
| slight | 27(31.4) | 19(30.7) | 8(33.3) |  |
| moderate | 26(30.2) | 20(32.3) | 6(25.0) |  |
| severe | 8(9.3) | 4(6.5) | 4(16.7) |  |
| extreme | 1(1.2) | 1(1.6) | 0(0.0) |  |
| **Anxiety or discomfort, n(%)** |  |  |  | 0.79 |
| Not anxious or depressed | 50(58.1) | 35(56.5) | 15(62.5) |  |
| Slightly | 25(29.1) | 17(27.4) | 8(33.3) |  |
| Moderate | 9(10.5) | 8(12.9) | 1(4.2) |  |
| Severe | 1(1.2) | 1(1.6) | 0(0.0) |  |
| Extreme | 1(1.2) | 1(1.6) | 0(0.0) |  |

*Exposed are participants with history of COVID-19 plus had COVID-19 test **OR** Anti-nucleocapsid antibody positive
